# Tumor-specific gene therapy for pancreatic cancer using human neural stem cells encoding carboxylesterase

**DOI:** 10.18632/oncotarget.12173

**Published:** 2016-09-21

**Authors:** Sung S. Choi, Kichul Yoon, Seon-A Choi, Seung-Bin Yoon, Seung U. Kim, Hong J. Lee

**Affiliations:** ^1^ Biomedical Research Institute, Chung-Ang University College of Medicine, Seoul, Korea; ^2^ Seoul Adventist Hospital, Seoul, Korea; ^3^ Department of Internal Medicine, Korea University College of Medicine, Seoul, Korea; ^4^ Futuristic Animal Resource & Research Center (FARRC), Korea Research Institute of Bioscience and Biotechnology (KRIBB), Ochang, Korea; ^5^ Division of Neurology, Department of Medicine, UBC Hospital, University of British Columbia, Vancouver, BC, Canada

**Keywords:** CPT-11, carboxyl esterase (CE), gene therapy, human neural stem cell, pancreatic cancer

## Abstract

Advanced pancreatic cancer is one of the most lethal malignant human diseases lacking effective treatment. Its extremely low survival rate necessitates development of novel therapeutic approach. Human neural stem cells (NSCs) are known to have tumor-tropic effect. We genetically engineered them to express rabbit carboxyl esterase (F3.CE), which activates prodrug CPT-11(irinotecan) into potent metabolite SN-38. We found significant inhibition of the growth of BxPC3 human pancreatic cancer cell line *in vitro* by F3.CE in presence of CPT-11. Apoptosis was also markedly increased in BxPC3 cells treated with F3.CE and CPT-11. The ligand VEGF and receptor VEGF-1(Flt1) were identified to be the relevant tumor-tropic chemoattractant. We confirmed *in vivo* that in mice injected with BxPC3 on their skin, there was significant reduction of tumor size in those treated with both F3.CE and BxPC3 adjacent to the cancer mass. Administration of F3.CE in conjunction with CPT-11 could be a new possibility as an effective treatment regimen for patients suffering from advanced pancreatic cancer.

## INTRODUCTION

Pancreatic cancer is one of the most lethal human cancers and continues to be a major health problem. Conventional therapeutic approaches, such as surgery, radiation, chemotherapy, or combinations of these modalities, have little impact on the course of pancreatic cancer [1]. Patients with locally advanced pancreatic cancer have a median survival time of 8–12 months, and patients with distant metastases have significantly worse outcomes with a median survival time of 3– 6 months [2–4]. The aggressive nature and disappointing treatment results of advanced pancreatic cancer requires novel approach.

The recent discovery of the inherent tumor-tropic properties of neural stem cells (NSCs) [5] has led to the development of enzyme-prodrug gene therapy approach for malignant tumors in the brain including gliomas and medulloblastomas [6–9]. NSCs could be transduced with therapeutic genes in high efficiency, and rapidly expand to numbers required for therapeutic applications. We have previously generated a clonally derived immortalized human NSC line via a retroviral vector encoded with v-myc gene, and the HB1.F3 (F3) human NSCs were utilized in stem cell-based therapy in animal models of human neurological disorders [10–13].

The use of therapeutic NSCs is highly attractive because delivery vehicles can disseminate therapeutic gene products to invasive cancer cells. In previous studies, we have transduced F3 human NSCs with cytosine deaminase (CD) which converts the prodrug 5-fluorocytosine (5-FC) to 5-fluoruracil (5-FU). Transplantation of F3.CD NSCs and administration of 5-FC in mice bearing brain tumors including glioma, medulloblastoma and brain metastases of breast cancer and lung cancer resulted in significant inhibition of tumor growth [6–8, 14–25].

More recently, we have transduced F3 human NSCs with carboxylesterase, an enzyme that hydrolyzes chemotherapy agent CPT-11 (Irinotecan) to SN-38 (7-Ethyl-10-hydroxy-camptothecin), which is 1000 times more potent than CPT-11. F3.CE NSCs was administered in mice bearing brain tumors and other solid cancers including neuroblastoma, melanoma and ovarian cancer, then administration of CPT-11 was followed. There was significant inhibition of cancer growth in cancer bearing animals. We established the proof of concept that various cancers including brain tumors and other solid cancers can be effectively targeted using this approach [19, 26–30]. In the present study, we evaluated the therapeutic efficacy of F3 human NSCs encoding carboxylesterase administered in combination with prodrug CPT-11 to nude mice bearing pancreatic cancer.

## RESULTS

### F3.CE human NSC line expressing rabbit carboxylesterase (CE)

**Figure 1:**
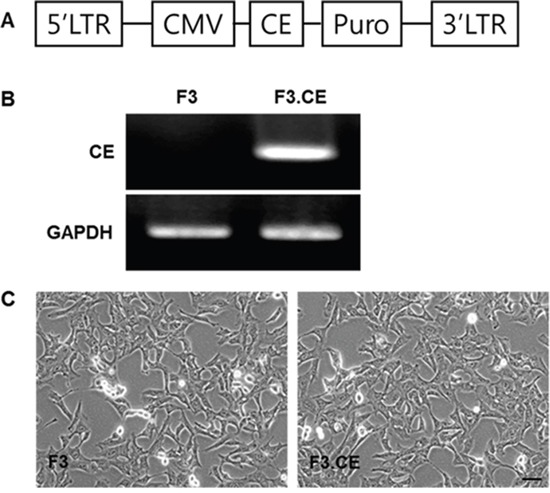
Establishment of HB1.F3 human neural stem cells expressing the carboxylesterase (CE) gene Expression of CE transcript was confirmed by RT-PCR, detecting CE transcripts in F3.CE, but not in F3 cells. **A.** Vector map of pLPCX.CE. **B.** Successful transduction of CE was confirmed by reverse transcription-PCR (237 bp). GAPDH = control. **C.** Phase contrast microscopy of F3 and F3.CE human NSCs. F3 : nerual stem cells, CE : carboxyl esterase, RT-PCR : reverse transcription-polymerase chain reaction

**Figure 2:**
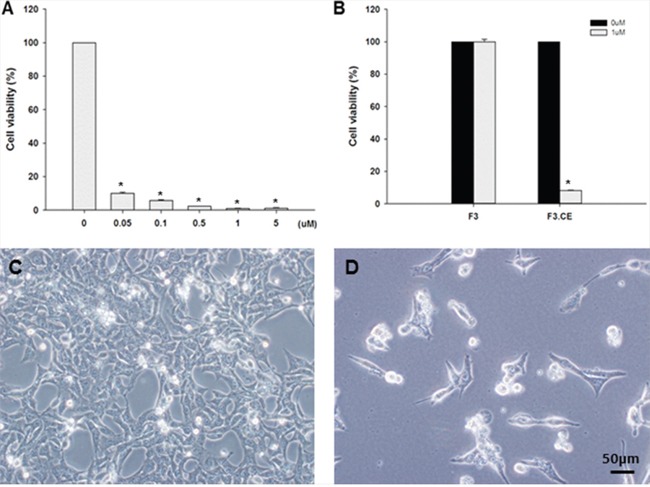
Suicide effect of F3.CE/CPT-11 system **A.** 1×10^4^ F3.CE cells were cultured with various concentration of CPT-11 at 37°C for 48 hr and the survival was determined. CPT-11 at concentration of 1 μM for 48 hrs killed >98% of F3.CE cells. **B.** BXPC3 human pancreatic cancer cells were incubated with 1 μM CPT-11 (3.75 mg/kg) for 48 hr with or without F3.CE cells. **C.**, **D.** BXPC3 cancer cells co-incubated with or without F3.CE cells for 48 hr.

Expression of CE gene in the F3.CE cells was analyzed by reverse transcription-PCR. CE transcript was detected in F3.CE cells but not in parental F3 cells (Figure 1). To confirm the enzyme activity of CE, F3.CE cells were exposed to CPT-11 at concentrations of 0.05-5 μM for 48 hr. The rate of survival in F3.CE cells was reduced considerably by 48 hr exposure to prodrug CPT-11 at concentrations of <5 μM (Figure 2). When co-culture of BxPC3 pancreatic cancer cells and F3.CE cells were exposed to 1 μM CPT-11 for 48 hr, less than 20% of BxPC3 cancer cells survived, indicating that F3.CE cells processed prodrug CPT-11 efficiently into cytotoxic SN-38 (Figure 2).

### Assessment of apoptosis of BxPC3 cells treated with F3 or F3.CE

**Figure 3:**
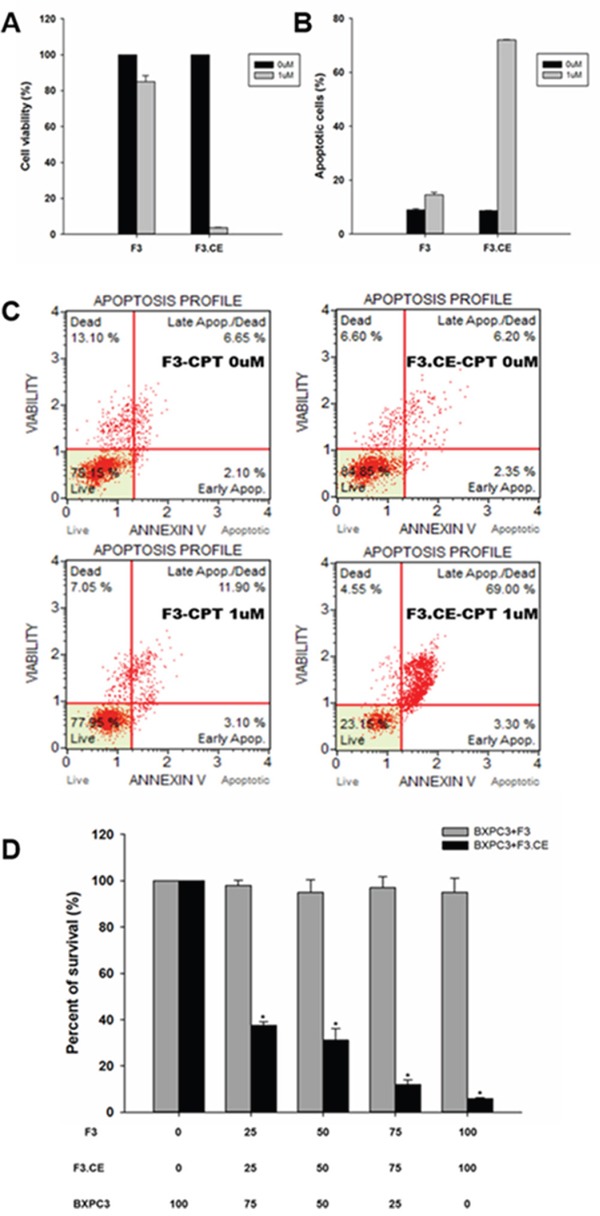
Assessment of apoptosis by BxPC3 cells were treated with F3 or F3.CE and CPT-11 BXPC3 cells were treated with or without F3.CE cells at the indicated doses for 48 hrs. **A.** Cell viability was measured with propidium iodine (PI). Significantly less percentage of BXPC3 cells were viable when treated with 1 μM CPT-11 and F3.CE, compared to F3. **B.**, **C.** annexin V-based apoptosis assay was performed as described in Materials and Methods. BXPC3 cells treated with CPT-11 and F3.CE showed significantly increased ratio of apoptotic cells. **D.** Bystander effect of the CE produced by the F3.CE cells was confirmed using a co-culture system of F3 or F3.CE cells and BXPC3 pancreas adenocarcinoma cells. BXPC3 cells with F3 or F3.CE cells were seeded in 96-well plates (total 1 × 10^4^ cells per well, BXPC3 cells: F3 or F3.CE cells = 100:0, 75:25, 50:50, 25:75, or 0:100). After 48 hrs in co-culture, cells were treated with 1.0 μM/mL CPT-11 for 48 hrs and cell survival was determined (each group, n = 3) (p< 0.05).

Cell viability of BxPC3 was measured with propidium iodine (PI)-labeled dead cells using Muse cell count and viability kit. Viable cells significantly decreased with F3.CE cells in the presence of 1uM CPT-11 (Figure 3A).

Annexin V-based apoptosis assay showed significant increase in the ratio of apoptotic cells in BxPC3 cells treated with F3.CE and CPT-11 (1 uM) at the same time. This assay solely measures apoptotic cells only, without counting necrotic cells. This phenomenon was not observed with F3 and CPT-11(1 uM), or with CPT-11(0 uM) (p<0.05) (Figure 3B and 3C).

### *In vitro* bystander effects on pancreatic cancer cells

Next, *in vitro* bystander effects of F3.CE cells on BxPC3 pancreatic cancer cells were determined using F3.CE - BxPC3 co-culture system and conditioned medium derived from F3 or F3.CE cells. In the co-culture experiment, application of 1 μM CPT-11 to BxPC3 pancreatic cancer cells had little effects on the survival until 48 hrs after the treatment. Toxic effects of CPT -11 (1 μM) was not observed when BxPC3 cancer cells were co-cultured with parental F3 cells (Figure 3D). In contrast, the survival of BxPC3 cancer cells co-cultured with F3.CE cells (cancer cells: F3.CE cells = 75:25, 50:50, or 25:75) was significantly reduced by 48 hr after exposure to 1 μM CPT-11 (P < 0.05, Figure 3D). Without CPT-11, co-culture with F3 or F3.CE had no effect on the survival of BxPC3 cancer cells (data not shown).

### *In vivo* therapeutic efficacy of F3.CE cells in cancer bearing mice

**Figure 4:**
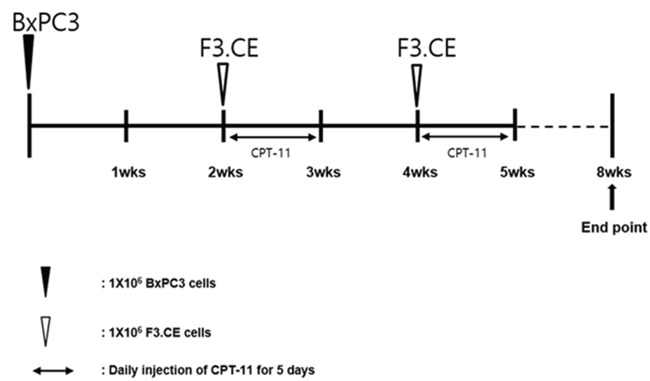
Timeline for the establishment of pancreas adenocarcinoma animal model and subsequent treatment using F3.CE cells and CPT-11 Human pancreas adenocarcinoma cells (1 × 10^6^ cells in 10 μL PBS) was injected into the subcutaneous dorsa of mice in the proximal midline. 6-week old SCID mice (n=7 each). At 14 and 28 days after tumor cell implantation, F3.CE cells (1 × 10^6^ cells in 100 μL PBS) were injected subcutaneously at four sites, 1 mm distant from the tumor. At 15~19 and 29~33 days after tumor cell implantation, CPT-11 (3.75 mg/kg) was injected into peritoneum once a day. Eight weeks from tumor implantation, the mice were sacrificed and the tumor mass measurement was performed.

**Figure 5:**
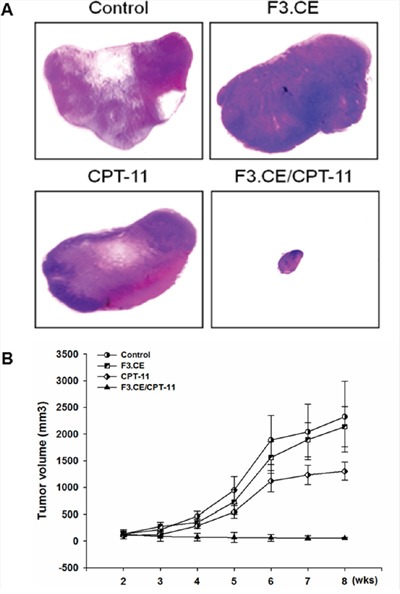
Treatment with F3.CE cells and CPT-11 has a significant therapeutic effect *in vivo* **A.** The representative images of skin slices for each group. Tumor bearing mice treated with F3.CE and CPT-11 showed significantly smaller tumor volumes compared with other groups including sham control group, tumor bearing animals transplanted with F3.CE cell but without CPT-11 treatment or animals injected with CPT-11 but without F3.CE cell transplantation (H&E stain). **B.** Tumor bearing mice treated with F3.CE and CPT -11 showed significantly smaller tumor volumes compared with other groups including sham control group, tumor bearing animals transplanted with F3.CE cell but without CPT-11 treatment or animals injected with CPT-11 but without F3.CE cell transplantation.

Timeline for the establishment of pancreas adenocarcinoma animal model and subsequent treatment using F3.CE cells and CPT-11 is shown on (Figure 4). In histologic study performed at 3 weeks after the last CPT-11 injection, cancer bearing animals treated with F3.CE cells and CPT-11 showed a significant reduction in cancer volume (Figure 5). The *in vivo* therapeutic efficacy of F3.CE cells against pancreas cancer was determined by tumor volume measurement. We measure and trace the tumor volumes from 2 weeks to end point at 8 weeks (Figure 5B). When final tumor volumes were determined 3 weeks after the last CPT-11 injection, the F3.CE + CPT-11 group mice showed significantly reduced tumor volumes (mean ± S.E. = 55.1 ± 15.8 mm^3^) compared with the sham control (2324.9 ± 662.8 mm^3^, p=0.001), F3.CE only group (2137.6 ± 377.5 mm3, p=0.001), and CPT-11 only group (1302.6 ± 168.6 mm3, p=0.001), respectively. There was 97.6 % reduction in tumor volume in F3.CE + CPT-11 group compared with the sham control group. There was 44% reduction in tumor volume in CPT-11 only group animals indicating that CPT-11 also acts as anticancer therapeutic. F3.CE cells encoding rabbit CE enzyme could convert chemotherapeutic agent CPT-11 into its more potent form, SN-38 at the site of the cancer and induced significantly additive tumor-killing activity.

## DISCUSSION

In the present study, we observed that human pancreatic cancer cell line BxPC3 treated with F3.NSCs expressing rabbit carboxylesterase (F3.CE) and application of prodrug CPT-11 showed marked growth inhibition and increased apoptosis *in vitro*. We also confirmed that transplantation of F3.CE and CPT-11 in immune-incompetent mice bearing BxPC3 human pancreatic cancer cells markedly inhibited cancer growth.

The NSCs were engineered to express rabbit carboxylesterase (CE). The enzyme activates the prodrug CPT-11 to the active drug SN-38, which has 1,000-fold more potent topoisomerase I inhibitory effect. In turn, SN-38 preferentially kills dividing cancer cells specifically at the tumor sites [30]. We have previously reported that F3.CE cells migrate selectively to tumor sites and they have a therapeutic effect on disseminated neuroblastoma [26, 30], melanoma [28], ovarian cancer [19], breast cancer brain metastasis [17] and medulloblastoma [29] upon administration of prodrug CPT-11. Recently we also have reported that the NSCs expressing the cytosine deaminase (CD). NSCs expressing CD and interferon-β selectively migrated toward cancer mass in nude mice bearing the PANC-1 human pancreatic cancer. Following administration of prodrug 5-fluorocytosine (5-FC), marked inhibition of cancer growth was observed [33].

Multimodal treatment for pancreatic cancer including surgical resection, radiation and chemotherapy has substantially improved the survival rate in patients with pancreatic cancer, however, it remains incurable in large proportion of patients. Therefore, there is profound need for both effective and less-toxic therapy for patients suffering from pancreatic cancer. The gene therapy approach targeting pancreatic cancer should fulfill this requirement.

Stem cell-based gene therapy using neural stem cells (NSCs) has received much attention as an innovative cancer therapy [11]. Previously we have adopted CE/CPT-11 enzyme/prodrug gene therapy approach for treatment of gliomas and disseminated neurobastoma animal models [19, 26–30] involving the conversion of a prodrug CPT-11 into SN-38 [30, 34]. CPT-11 (Irinotecan) is known to induce severe toxicities (diarrhea, neutropenia) that limit its clinical use [34]. The complex pharmacokinetics of CPT-11 and the involvement of several enzymes other than UGT (i.e., carboxylestherases, CYP450 isoforms), and transmembrane transporters (ABCB1, ABCC1, ABCG2, SLCO1B1) make it difficult to identify patients with an optimal sensitivity and specificity [35]. However, if our approach of activating CPT-11 with the designated enzyme inside of the tumor using NSCs could be used clinically, it might strengthen the efficacy and safety of the chemotherapy.

In conclusion, the present results demonstrate for the first time that the F3.CE cells successfully exerted therapeutic efficacy on BxPC3 pancreatic adenocarcinoma cells upon administration of CPT-11.

## MATERIALS AND METHODS

### Cell culture

HB1.F3 (F3), a stably immortalized human NSC cell line, was derived from human fetal telencephalon at 15 weeks of gestation by introducing a retroviral vector encoding v-myc [31, 32]. F3 and F3 cells overexpressing rabbit CE gene (F3.CE) cells were cultured in Dulbecco's modified Eagle's medium (DMEM) supplemented with 10% fetal bovine serum (FBS), 2 mmol/l L-glutamine, 100 units/mL penicillin and 100 μg/mL streptomycin (DMEM-10% FBS) (Sigma-Aldrich, St. Louis, MO). BxPC3 human pancreas adenocarcinoma cell line was obtained from Dr. Yun-Hee Kim in the National Cancer Center (South Korea) and maintained in DMEM-10% FBS.

### Generation of F3.CE human NSC line

The clonal F3.CE human NSC line was derived from the parental F3 NSC line. An expression plasmid encoding rabbit CE was constructed using the retroviral pLPCXpuro (Clontech, Palo Alto, CA) as previously described [30]. Successful transduction of the F3.CE cells was confirmed by reverse transcription–PCR (Figure 1) using the primer pair shown in Table 1. To confirm the activity of CE in F3.CE cells, the cytotoxic effect of CPT-11 on F3 or F3.CE cells was analyzed using a cell viability assay. F3 or F3.CE cells (3 × 10^4^/well) were plated in 6-well plates. Twenty-four hrs after seeding, 0.5-5 μM of CPT-11 (Hanmi Pharma, Seoul, Korea) was applied for 48 hrs, and the status of the cells was analyzed using a microscope. Cell viability was performed using Muse™ Cell Analyzer (Millipore, Billerica, MA) following manufacturer's instruction. Briefly, after the indicated treatments, the cells were detached and washed with PBS and incubated with Muse cell count and viability solution for 5min. After staining, the cells were processed in muse apparatus.

**Table 1:** Sequence of PCR primers

Gene	Sequence	Size (bp)
SCF	Sense 5'-ACTTGGATTCTCACTTGCATTT-3' Antisense 5'-CTTTCTCAGGACTTAATGTTGAAG-3'	505
c-kit	Sense 5'-GCCCACAATAGATTGGTATTT-3' Antisense 5'-AGCATCTTTACAGCGACAGTC-3'	332
SDF-1	Sense 5'-ATGAACGCCAAGGTCGTGGTC-3' Antisense 5'-GGCTGTTGTGCTTACTTGTTT-3'	200
CXCR4	Sense 5'-CTCTCCAAAGGAAAGCGAGGTGGACAT-3' Antisense 5'-AGACTGTACACTGTAGGTGCTGAAATCA-3'	733
VEGF	Sense 5'-AAGCCATCCTGTGTGCCCCTGATG-3' Antisense 5'-GCTCCTTCCTCCTGCCCGGCTCAC-3'	541
VEGFR1	Sense 5'-GCAAGGTGTGACTTTTGTTC-3' Antisense 5'-AGGATTTCTTCCCCTGTGTA-3'	512
VEGFR2	Sense 5'-ACGCTGACATGTACGGTCTAT-3' Antisense 5'-GCCAAGCTTGTACCATGTGCG-3'	438
GAPDH	Sense 5'-CATGACCACAGTCCATGCCATCACT-3' Antisense 5'-TGAGGTCCACCACCCTGTTGCTGTA-3'	450

### *In vitro* “bystander effect” experiments

BxPC3 human pancreas adenocarcinoma cells were plated in 6-well plates with F3 or F3.CE cells (BxPC3 cells:F3 or F3.CE cells = 100:0, 75:25, 50:50, 25:75, or 0:100). BxPC3 and F3 or F3.CE cells were maintained in DMEM-10%FBS. After 24 hrs of cell growth, 1.0 μM CPT-11 was added to the mixed cell cultures and 48 hrs later, cell viability was determined utilizing Muse™ Cell Analyzer as described above.

### Apoptosis assay

Annexin V & Dead Cell Assay was performed utilizing Muse™ Cell Analyzer following manufacturer's instruction. Briefly, after the indicated treatments, the cells were incubated with Annexin V and Dead Cell Reagent (7-AAD) for 20min and the events for dead, late apoptotic, early apoptotic, and live cells were counted.

### Pancreas cancer animal model

Animal experiments in this study have been reviewed and approved by the Animal Care and Use Committee of Chung-Ang University (IRB: 11-0086). Six-week-old male BALB/c nude mice were purchased from Saeronbio Inc. (Kyunggi, Korea). Mice were caged in a barrier care facility, and fed with animal chow and water ad libitum. Mice were randomized into four groups, 7 mice each. The control group was injected with BxPC3 human pancreatic adenocarcinoma cells only. The second group received BxPC3 cancer cells and prodrug CPT-11. The third group received BxPC3 cancer cells and F3.CE human NSCs, while the fourth group receiving BxPC3 cancer cells and F3.CE human NSCs was treated with CPT-11. The fourth group was administered with F3.CE and CPT-11 regimen according to the time line (Figure 4).

Animals were anesthetized via intraperitoneal injection of Zoletil before all surgical procedures and were observed until fully recovered. Before tumor cell injection, mice were shaved and the dorsal skin cleaned with ethanol. Human pancreas cancer cells (BxPC3, 1 × 10^6^ cells in 100 μL PBS) were injected into the subcutaneous dorsa of mice in the proximal midline of 6-week old SCID mice (n=7). At 14 and 28 days after cancer cell implantation, F3.CE cells (2.5 × 10^5^ cells in 25 μL PBS) were injected subcutaneously at four sites (i.e. 1 × 10^6^ cells in 100 μL PBS in total), 1 mm distant from the cancer. At 15~19 and 29~33 days after cancer cell implantation, CPT-11 (3.75 mg/kg) was injected into peritoneum once a day. Eight weeks after cancer implantation, the mice were sacrificed in accordance with institutional guidelines, and the removed cancer tissues were fixed in 4% paraformaldehyde and the cancer mass measurement was performed.

### Immunohistochemistry

Representative cancer tissues (three from each group) were paraffin-embedded. Sections (5 μm) were stained with hematoxylin-eosin (H&E) to evaluate tumor size.

### Statistics analysis

All experiments is repeated three times. Two-way ANOVA tests were used to evaluate differences in all experiments between groups. P values < 0.05 were considered statistically significant. Analyses were performed using SigmaPlot version 12; (Systat software Inc., San Jose, CA, USA).
